# Female Sexual Dysfunction Among a Sample of Egyptian Patients with Asthma

**DOI:** 10.2174/1874306402014010038

**Published:** 2020-10-23

**Authors:** Mona Reda, Dina Ruby

**Affiliations:** 1 Department of Psychiatry, Ain Shams University, Cairo, Egypt; 2 Department of Chest Diseases, Ain Shams University, Cairo, Egypt

**Keywords:** Asthmatic females, Female sexual dysfunction (FSD), Quality of life, World health organization, Egyptian females, Healthy females

## Abstract

**Background:**

Despite asthma being a worldwide disease, still little awareness regarding the sexual function of asthmatic patients exists. So this study attempts to assess the Female Sexual Dysfunction (FSD) amongst Egyptian females with asthma and its burden on their quality of life.

**Materials & Methods:**

The sample consisted of 180 subjects, comprising 90 asthma patients and 90 healthy controls aged between 20 - 45 years old, who visited the Chest Department Outpatient Clinic of Ain Shams University Hospital between January and December 2018. We reported all the subjects' demographic and clinical data; both groups answered an Arabic version of the Female Sexual Function Index (Ar FSFI) and World Health Organization Quality of Life Questionnaire abbreviated version (WHOQL-Bref).

**Results:**

90% of asthmatic females had FSD; total Female Sexual Function Index score was 12.956 ± 10.3 in asthmatic females compared to 25.423 ± 5.521 in healthy controls; 45.6% of asthmatic females with sexual dysfunction had moderate asthma and 86.4% had uncontrolled asthma, 40.1% of them had a low educational level and 80.2% were unemployed.

**Conclusion:**

Jobless females with severe uncontrolled asthma and a low educational level had higher sexual dysfunction and a poor quality of life.

## 1 INTRODUCTION

Asthma is defined as the chronic inflammation of the airway with respiratory symptom history that varies in its intensity over time along with variable expiratory airflow limitation. Diagnosis depends on respiratory symptom history and the confirmation of variable expiratory airflow limitation [1].

Asthma is triggered by a variety of factors such as allergen or irritant exposure, weather changes, exercise and infections [2]. Sex can also stimulate an asthma exacerbation in different ways, such as human seminal plasma allergy, post-coital asthma, condom-induced asthma [3]. Sexual intercourse can trigger severe asthma exacerbations that need emergency care, hospital admission or even mechanical ventilation [4]. The association between asthma and sexual activity has been described before. Few reports have addressed this issue [5]; however, little is known regarding sexual problems among married Egyptian women [6]. Asthma in females is an important public health issue as asthma changes from being predominant in boys in early adolescence to more predominant in girls due to hormonal causes; so women carry the higher burden of morbidity and mortality due to asthma [7].

Clinicians rarely discuss sexual matters with women [8], and nurses who are responsible for offering sexual counseling rarely do this in their practice [9]. This is due to lack of education, time, knowledge and awareness; they may also feel uneasy to discuss this issue or fear embarrassing the patient. On the other hand, patients may refuse to discuss problems related to their sexual life [10]. Therefore, sexual limitation continues to be a part of the asthma-related quality of life issues, that is often missed both in clinical practice and research [11] as it goes unnoticed, and ends up causing psychiatric illnesses and social behaviors that may be unacceptable to the society [2]. So the study was designed to assess Female Sexual Dysfunction (FSD) among Egyptian females with asthma and its burden on their quality of life.

## 2 MATERIALS AND METHODS

This was a cross-sectional study conducted between January to December 2018 after obtaining the approval of the Ethical Committee of the Faculty of Medicine at Ain-Shams University under the approval no. FMASU R 69/2018.

The targeted sample size in each group was calculated [12]; one-hundred & eighty of 250 consecutive married females aged between 20 to 45 years were divided into two groups. The first group comprised asthmatic females visiting the Chest Department Outpatient Clinic of Ain-Shams University Hospital for a follow-up visit. The second group included healthy females and were considered as the control group; these were visitors or employees in the same participating Department during the same time interval.

The inclusion criteria of both groups were married females ≥ 20 years of age, who can read and write. In the asthmatic group, females were diagnosed as having bronchial asthma according to GINA 2018 [1], and stable at the time of inclusion (no exacerbations during the four weeks prior to their inclusion in the study). The exclusion criteria for both groups included those with any chronic illness *i.e*., Diabetes, IHD, a medical history of depression, medical history of gynecological disorders, or abuse of drugs that may cause sexual dysfunction, pregnant females and illiterate ones.

Oral informed consent was received from all participants by the Research Team before questionnaire distribution. After explaining its aim, their permission was obtained to use their answers for statistical analysis. Then the patients were interviewed with the help of the Inpatient Chest Department sheet including sociodemographic data for both groups such as age, total years of marriage, number of offspring, educational level, occupational status, medical history and health problems, obstetrical and gynecological history and comorbidities; drug history and case history for the asthmatic group included the age of asthma onset, duration of asthma, asthma severity level and asthma control level according to GINA 2018 [1]. Both groups filled two questionnaires: Female Sexual Function Index (FSFI) [13] Arabic Version and the World Health Organization Quality of Life Questionnaire abbreviated version (WHOQoL-Bref) [14] in Arabic.

The Female Sexual Function Index (FSFI) [15] was translated to Arabic by Anis *et al*. [13] It is a self-administered questionnaire; 19 different questions are required to be answered from a six possible answers multiple-choice questionnaire, provided that answers for each item best defines/describes the female condition over the last four weeks. The lower the score, the higher the chance of having FSD [13]; the optimal cutoff score for FSD was 26.55 for the total score [16] and this was applied in the present study.

The WHOQoL-Bref [17] was translated to Arabic by Abdel Hai *et al*. [14]. It is a self-administered questionnaire and consists of 26 items; two items assess the overall quality of life and satisfaction with health. The remaining 24 items are divided into four domains (physical, psychological, social, and environment); all questions concerned the last two weeks of the patients and were graded on a 5 point scale [17].

## 3 STATISTICAL ANALYSIS

Quantitative variables were presented as mean and standard deviation. Comparison between means was done through the independent t-test. Categorical data were presented as count and percentages. A comparison between proportions was done using the chi-square test. P values equal to 0.05 or less were considered statistically significant. Data analysis was done by SPSS version 20.

## 4 RESULTS

The present study was carried out in the Chest Department Outpatient Clinic at Ain-Shams University Hospital between January and December 2018. The total sample size was 250 subjects; fifty females refused to share related outcomes in the study and twenty returned questionnaire with missing data, with a response rate of 72%, so 180 subjects were divided into two groups, the asthmatic females group (n=90) and the healthy female group as control (n=90). The mean age of our study group was 37± 7 years in asthmatic patients and there was no statically significant difference between both groups with regard to other socio-demographic data shown in Table **1**.

In the asthmatic group, the mean age of asthma onset was 28 ± 9 years, and the mean asthma duration was 9 ± 6 years; more than half of the asthmatic group was uncontrolled, 41.1% of them had moderate asthma while 36.6% had severe asthma, and the remaining 22.2% had mild asthma.

**Fig. (1):**
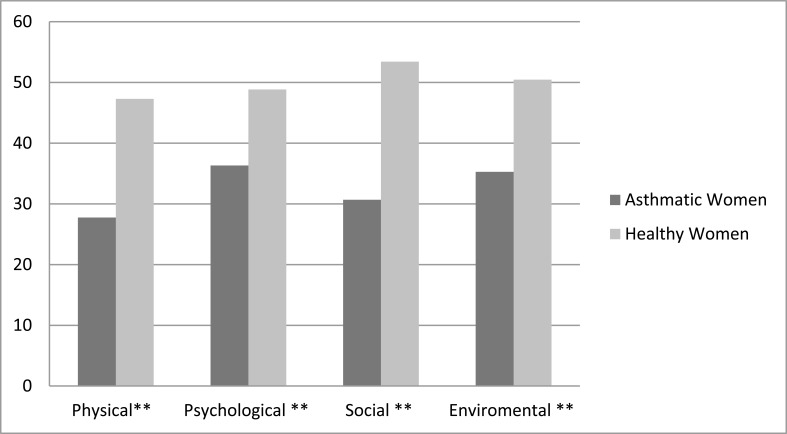
Comparing the Quality of Life (WHOQOL-BREF) between the asthmatic and healthy female. (WHOQOLBREF: World Health Organization Quality of Life Questionnaire abbreviated version, **: highly significant at p value <0.01)

The FSFI total score in the asthmatic group was12.956 ±10.300, while the healthy group was 25.423± 5.521. The asthmatic group also had a low score in all subsets of FSDI shown in Table **2** and in the WHOQOL-BREF four domains (Fig. **1**).

According to the optimal cut off scores for FSD [17], we found that FSD was 90% (n= 81) in asthmatic females and 47% (n= 43) in the healthy control sample, even after adjusting the confounding factors (age, number of offspring, number of years of marriage, education, occupational status). As shown in Table **3**, when we compared asthmatic and healthy females with respect to sexual dysfunction, we found that asthmatic patients had lower scores in all domains of FSFI. Furthermore, as Fig. (**2**) presents, asthmatics had lower scores in all domains of QOL.

**Fig. (3):**
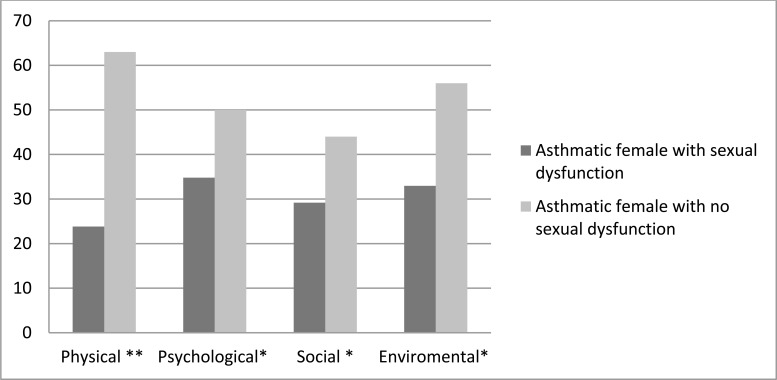
Comparison of the Quality of Life (WHOQOL-BREF) between asthmatic female with and without sexual dysfunction (WHOQOLBREF: World Health Organization Quality of Life Questionnaire abbreviated version,*: Significant at p value <0.05 level. **: highly significant at p value <0.01)

As shown in Table **4**, according to FSD, we divided asthmatic patients into two groups: asthmatic with Sexual Dysfunction (SD) and asthmatic without SD. It was found that asthmatic females with SD had less children and lower educational level (40.1% completed only secondary education), 80.2% were unemployed, 45.6% of them had moderate asthma and 86.4% had uncontrolled asthma and poorer quality of life in all QOL domains (Fig. **3**).

## 5 DISCUSSION

Sexuality is a fundamental component of quality of life for both genders which reflects their level of physical, psychological and social well being [18]. In Egypt, together with other Arab world societies, discussing sexual matters is prohibited for cultural reasons; this has led to reluctance in seeking medical help for sexual problems [8].

Chronic and Pulmonary diseases, in particular, can increase the incidence of sexual dysfunction in both genders [12, 19, 20]. Medications used to improve their condition can also impair sexual function [10]. But in chronic obstructive pulmonary disease (COPD) and asthma patients inhaling steroids only, minimal systemic effects and no decrease of sex steroid hormone have been observed. Therefore, the medication plays no role in the pathophysiology of the FSD of previous diseases [21].

Assessment of sexual function and quality of life performed in this study revealed that asthmatic patients had worse sexual function than healthy ones throughout all sexual function domains, with also low parameters of all QOL domains even after matching most of the factors that may influence the results including age, education level, occupational status, years of marriage and number of children in both groups.

Several studies have emphasized the effect of asthma on patients’ sexual life, especially among females, as Meyer *et al*. [11] stated that 58% of patients' sex life was affected by asthma. Kaptein *et al*. [22] also found that patients were afraid of dyspnea during their sexual intercourse, and still, one out of seven talked to their physician about the problem. Therefore, the result of this study was found to match with former studies.

The age of our studied patients was not a risk factor for SD and that was inconsistent with the study by Meyer *et al*. [11]; this can be explained by the fact that as our studied sample was young, the mean age of the studied asthmatic females was 37 years and Meyer *et al*. [11] determined patients in their 40’s and the over-age group experiences sexual affection about three times more than young people.

By reviewing available literature on this topic [11, 18, 23], it was found that there were several independent factors such as educational level, occupational status, asthma duration, severity and control level of asthma, all of which can affect female sexual life and this was consistent with our study.

Asthmatic patients with long duration of illness have been proven to have more SD [23-26] due to psychiatric and systemic complications that appear because of prolonged disease duration [24], as evident in the current study. Educational level has also been found in previous studies to play a key risk factor in developing SD in Asthmatics [23-27], which was also consistent with our findings as about 70% of the SD asthmatic sample was found to have either complete primary or secondary education only. This may be because patients with low education lack the knowledge about sexuality and consider it a taboo, accepting the condition with the conception of powerlessness [23].

Some studies [11, 23] have found that unemployment status can lead to emotional stress, exacerbating asthma and can induce the economic burden on patients and lead to non-compliance with asthma treatment, while in turn leading to SD. This was also found in this study as 80% of asthmatics in the current study were found to be jobless.

Various studies [28-30] revealed that quality of life is negatively affected by chronic diseases. The former studies [11, 23, 24] also found that asthma can lead to sexual limitations, that in turn, can affect the quality of life, which has also been proven in this study as asthmatic females with SD had lower parameters of QOL domains.

This study’s limitation accounts for the small sample size attributed to that sexuality is perceived as something confidential and people do not want to talk about it.

## CONCLUSION

90% of the patients with asthma experienced sexual dysfunction and had poorer quality of life. Education level, occupational status, asthma duration, asthma severity and control level were independent risk factors for sexual dysfunction and poor quality of life.

Therefore, Health education must be offered for asthmatic females by Physicians and nurses through discussing more comfortable sexual positions for their respiratory condition to achieve satisfaction in their sexual life without the fear of breathing difficulties. As the aim of the treatment for asthma is not only to improve the lung function but also to improve the quality of their life.

## Figures and Tables

**Fig. (2):**
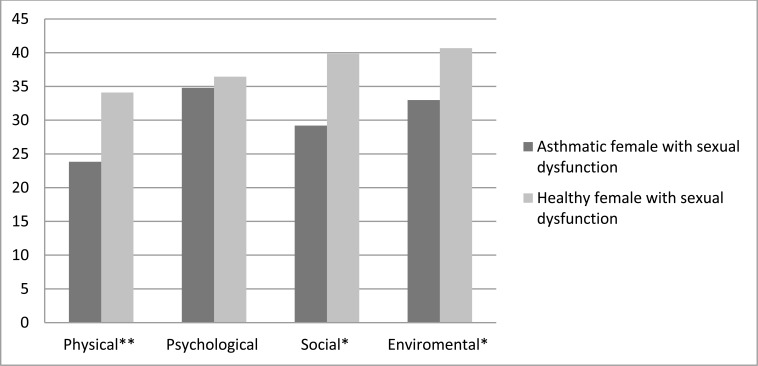
Comparison of the Quality of Life (WHOQOL-BREF) between asthmatic and healthy female with sexual dysfunction (WHOQOLBREF: World Health Organization Quality of Life Questionnaire abbreviated version,*: Significant at p value <0.05 level. **: highly significant at p value<0.01)

**Table 1:** Sociodemographic characteristics between asthmatic and healthy females.

-		** Groups**				
		** Asthmatic Females**		** Healthy Females**		**P- value**
Age (years)	Mean ±SD	37.044** ±**7.234		35.544** ± **4.485		0.096
Number of years of marriage	Mean ±SD	15.844 ± 6.669		15.144** ±** 6.098		0.463
Number of children	Range	1 - 3		1 - 4		0.867
	Mean ±SD	3** ±** 1		3 **±** 1		
Education	Primary	N=24	26.67%	N=22	24.44%	0.286
	Secondary	N=33	36.67%	N=25	27.78%	
	High education	N=33	36.67%	N=43	47.78%	
Occupational Status	Not working	N=65	72.22%	N=59	65.56%	0.334
	working	N=25	27.78%	N=31	34.44%	

N: number. SD: standard deviation; T-Test or Chi-Square were used for statistical analysis

**Table 2:** Comparison of FSFI between the asthmatic and healthy females.

-		**Groups**		
		**Asthmatic Females**	**Healthy Females**	**P- Value**
Desire	Mean ±SD	2.347 **± ** 1.140	3.456** ±** 0.986	<0.001**
Arousal	Mean ±SD	1.983 **±** 1.881	3.961 **±**1.204	<0.001**
Lubrication	Mean ±SD	2.297 **±**2.1888	4.814** ±**1.235	<0.001**
Orgasm	Mean ±SD	2.182** ±** 2.099	4.039 **±**1.512	<0.001**
Satisfaction	Mean ±SD	2.467**±**1.729	4.556** ±**1.295	<0.001**
Pain	Mean ±SD	1.676**±**1.593	4.586** ±**1.247	<0.001**
Total score	Mean ±SD	12.956** ±**10.300	25.423**±**5.521	<0.001**

FSFI: female sexual function index. SD: standard deviation. **: highly significant at p value <0.01

T-Test was used for statistical analysis

**Table 3:** Comparing asthmatic and healthy females with respect to sexual dysfunction taking into account the sociodemographic data and FSFI.

-		**Groups**				**P- value**
		**Asthmatic female with Sexual dysfunction**		**Healthy female with sexual dysfunction**		
**Age (years)**	**Mean ±SD**	36.938** ±**7.623		35.233** ± **4.485		0.178
**Number of years of marriage**	**Mean ±SD**	15.827 ** ±** 7.033		14.930** ±** 5.954		0.478
**Number of children**	**Range**	1 - 3		1 - 4		0.409
	**Mean ±SD**	2**±** 1		2**±**1		
**Education**	**Primary**	29.63%	N=24	27.91%	N=12	0.221
	**Secondary**	40.74%	N=33	27.91%	N=15	
	**High education**	29.63%	N=24	44.19%	N=19	
**Occupational status**	**Not working**	80.25%	N=65	72.09%	N=31	0.301
	**working**	19.75%	N=16	27.91%	N=12	
**Desire**	**Mean ±SD**	2.074**± **0.833		3.063**±** 0.856		<0.001**
**Arousal**	**Mean ±SD**	1.670**±**1.715		3.128**±**1.121		<0.001**
**Lubrication**	**Mean ±SD**	1.985**±**2.084		4.153**±**1.397		<0.001**
**Orgasm**	**Mean ±SD**	1.802**±**1.855		2.856**±**1.296		0.001*
**Satisfaction**	**Mean ±SD**	2.163**±**1.546		3.912**±**1.392		<0.001**
**Pain**	**Mean ±SD**	1.462**±**1.536		4.077**±**1.454		<0.001**

FSFI: female sexual function index .N: number. SD: standard deviation. *: Significant at p-value <0.05 level. **: highly significant at p-value <0.01. T-Test or Chi-Square were used for statistical analysis

**Table 4:** Comparing asthmatic females without and with sexual dysfunction taking into account the sociodemographic data and asthma characteristics.

-		**Groups**					** P- value**
		**Asthmatic females with no sexual dysfunction**			**Asthmatic females with sexual dysfunction**		
**Age** (Years)	**Mean ±SD**	38.000** ± **0.000			36.938** ±**7.623		0.679
**Number of years of marriage**	**Mean ±SD**	16.000** ±** 0.000			15.827 **±** 7.033		0.942
**Number of children**	**Range**	3 - 3			1 - 3		0.024*
	**Mean ±SD**	3 **±** 0			2 **±** 1		
**Education**	**Primary**	N=24	0.00%		N=0	29.63%	<0.001**
	**Secondary**	N=33	0.00%		N=0	40.74%	
	**High education**	N=24	100%		N=9	29.63%	
**Occupational status**	**Not working**	N=65	0.00%		N=0	80.25%	<0.001**
	**working**	N=16	100%		N=9	19.75%	
**Duration of asthma (years)**	**Mean ±SD**	2.222**±** 0.441			9.765**± **5.297		<0.001*
**Age of onset of asthma (years)**	**Mean ±SD**	35.778**±**0.441			27.210**±**8.898		0.005*
**Asthma severity**	**Mild**	N=11		100%	N=9	13.58%	<0.001**
	**Moderate**	N=37		0.00%	N=0	45.68%	
	**Severe**	N=33		0.00%	N=0	40.74%	
Asthma control level	**Partly controlled**	N=11		100%	N=9	13.58%	<0.001**
	uncontrolled	N=70		0.00%	N=0	86.42%	

N: number. SD: standard deviation. *: Significant at p-value <0.05 level. **: highly significant at p-value <0.01. T-Test or Chi-Square were used for statistical analysis

## ABBREVIATIONS

FSD: = Female Sexual Dysfunction
FSFI: = Female Sexual Function Index
SD: = Sexual Dysfunction
WHOQOLBREF: = World Health Organization Quality of Life Questionnaire abbreviated version.
